# THE GENDERED LONG ARM OF CHILDHOOD: EARLY PARENT–CHILD RELATIONSHIP AND LATER-LIFE MENTAL HEALTH IN RURAL CHINA

**DOI:** 10.1093/geroni/igae098.3424

**Published:** 2024-12-31

**Authors:** Jia Wang, Mingyue Ma

**Affiliations:** The Hong Kong Polytechnic University, Hong Kong, Hong Kong; The Hong Kong Polytechnic University, Hong Kong, Hong Kong

## Abstract

The large body of literature examining adverse childhood experiences (ACE) on late-life health has paid relatively little attention to gender variation. We contend that a gender perspective is crucial for understanding if women’s disadvantages in health have an early origin from childhood. In particular, we suggest that non-material aspects of ACE, such as poor parent-child relationships, are more likely to exhibit gendered patterns in their influences on health, particularly in developing countries with traditional gender norms and higher gender inequality. Using five waves of data from the China Health and Retirement Longitudinal Study (CHARLES), we examine the associations between parent-child relationships in childhood and later-life mental health among Chinese men and women aged 45 and above in rural areas. Our analyses yield three important findings: 1) Compared to rural men, rural women tend to exhibit worse mental health in terms of both the baseline level and the developmental trajectory of mental health over time; 2) From a snapshot perspective, poorer parent-child relationship during early life is related to worse baseline level of mental health among both rural men and women, and this association is more pronounced among rural women; 3) From a temporal perspective, poorer parent-child relationship in early life is associated with higher likelihoods of experiencing depression throughout the whole survey period, particularly among rural women. Our study sheds new light on the significance of a gender lens when examining the early-life origins of health inequality.

